# Effects of a mutual recovery intervention on mental health in depressed elderly community-dwelling adults: a pilot study

**DOI:** 10.1186/s12889-016-3930-z

**Published:** 2017-01-03

**Authors:** Chao Wang, Yujie Hua, Hua Fu, Longfeng Cheng, Wen Qian, Junyang Liu, Paul Crawford, Junming Dai

**Affiliations:** 1 School of Public Health, Health Communication Institute, Fudan University, Shanghai, China; 2 National Institute of Occupational Health and Poison Control, Chinese Center for Disease Control and Prevention, Beijing, China; 3 Suzhou Center for Disease Control and Prevention, Suzhou, China; 4 Chongqing Center for Disease Prevention and Control, Chongqing, China; 5 School of Nursing, Midwifery and Physiotherapy, The University of Nottingham, Nottingham, UK; 6 Professorial Fellow at the Institute of Mental Health, Nottingham University, Nottingham, UK

**Keywords:** Mental health, Depression, Well-being, Intervention, Elderly population

## Abstract

**Background:**

The prevalence of depression in the elderly is growing worldwide, and the population aging in China makes depression a major health problem for the elderly adults and a tremendous burden to the society. Effective interventions should be determined to provide an approach solving the problem and improving the situation. This study examined the effectiveness of a mutual recovery program intervention on depressive symptom, sleep quality, and well-being in community-dwelling elderly adults with depressive symptom in Shanghai.

**Methods:**

Recruitment was performed between July 2012 and August 2012. Using a cluster randomized wait-list controlled design, we randomized 6 communities (*n* = 237) into either the intervention group (3 communities, *n* = 105) or to a wait-list control group (3 communities, *n* = 132). All participants met the inclusion criteria for depression, which were defined by The Geriatric Depression Scale (GDS-15). From March to May of 2013, participants in the intervention group underwent a 2-month mutual recovery program intervention. The intervention included seven 90-min, weekly sessions that were based on a standardized self-designed schedule. Depression was used as primary outcome at three measurement moments: baseline (T1), before intervention at 24 weeks (T2), and immediately after intervention at 32 weeks (T3). Well-being and sleep quality were used as the secondary outcomes, and were evaluated based on the WHO-5 Well-being Index (WHO-5) and the Self-administered Sleep Questionnaire (SSQ). Finally, a total of 225 participants who completed all the sessions and the three measurements entered the final analysis. Mixed-model repeated measures ANOVAs were performed to estimate the intervention effects.

**Results:**

There was no significant difference in gender, marriage, age structure, post-work type, and education background between the intervention and control group at baseline. Multivariate ANOVAs showed that there was no significant difference within the groups in terms of sleep, well-being, and depression at baseline and before the intervention. Mixed-model repeated measures ANOVAs detected a group × time interaction on depression, sleep, and well-being and showed a favorable intervention effect within groups immediately after the intervention.

**Conclusions:**

The mutual recovery program could be a creative and effective approach to improve mental health in older community-dwelling adults with depressive symptom.

**Electronic supplementary material:**

The online version of this article (doi:10.1186/s12889-016-3930-z) contains supplementary material, which is available to authorized users.

## Background

The elderly (8.87% for those aged 65 and over in 2010) are becoming one of the largest segments of the Chinese population. They already number 110 million, representing the largest elderly population in the world [1, 2]. In addition, major depression disorders are common among older adults and costs almost 80 billion dollars per year in China [3]; approximately, 10% elderly community-dwelling residents and 15% to 25% of hospitalized patients develop major depression [4]. This percentage would reach 35% with the inclusion of mild depression [5]. According to World Health Organization data, depression now accounts for the fourth-largest disease cost burden in the world and will be the second in 2020 [6–8].

Cognitive behavior therapy (CBT) is a popular psychotherapy which was originally designed to treat depression, and now commonly used to treat a wide range of mental disorders [9]. CBT is based on Beck’s (1979) cognitive theory of depression and aims to correct the faulty or maladaptive cognitive thinking and lead to changes in both behavior and affect. Other strategies such as exercise program, social interaction promotion, and relaxing techniques are often integrated into the CBT, which could help decrease depressive symptoms [10]. Studies have shown that CBT can effectively improve the symptoms of depression and is comparable in effectiveness to antidepressants and interpersonal or psychodynamic therapy [11]. However, its effectiveness for depression in older people is still mixed [12]. In traditional CBT, therapists take control of the entire process, which may lead CBT to lack interaction and communication between participants, as well as between participants and facilitators [13]. Elderly adults usually have difficulty in acquiring knowledge with lower level of education and slower reactions, whereas they usually like chatting together and sharing with each other, so traditional CBT may not be suitable for older depression sufferers. Moreover, access to face to face CBT is relatively limited because its delivery mode requires adequate therapist time and effort per treatment [14].

Facing the growing burden of depression, creative practice are needed to overcome the shortcomings as described in the previous study [15]. Mutual recovery is a comprehensive and inexpensive approach for mental health problems [16]. In mutual recovery, participants instead of facilitators may lead the intervention process. Participants in the mutual recovery group are encouraged to interact and communicate the knowledge taught in the sessions with each other as well as any other interesting topic not covered during the session. Through this process, social connections will be established and strengthened, and social support will be improved [17]. This kind of mutuality or reciprocity of mutual recovery will benefit everyone who is involved in the process of intervention [18–20]. As many commentators have remarked that mental health is often consolidated in strongly individualistic terms [21], mutual recovery opens up new possibilities for patient benefit by sharing individual practices and thoughts. Moreover, facilitators also have opportunities to receive feedback about the intervention and refine the schedule to produce a better outcome. In addition, music and story sharing, which create a supportive environment, can be used as an icebreaking tool for expressing experiences and emotions and persuading new and better identities and communities [22, 23]. The advantages of the main types of mental health intervention, such as CBT [11], problem solving [24], physical activity [25, 26], and relaxing therapy, can also be utilized in the mutual recovery session. Besides, the delivery mode of this directive group therapy is more efficient and cost-effective than traditional face to face intervention.

In this study, we developed a mutual recovery intervention based on the Chinese culture and conducted a series of training courses to in communities in Pudong District, Shanghai. The aim of this study was to examine the effectiveness of a mutual recovery intervention for elderly community-dwelling adults with depressive symptom. We hypothesized that our mutual recovery program would reduce elderly adults’ depressive symptoms and consequently improve their sleep quality and well-being.

## Methods

### Design

**Fig. 1:**
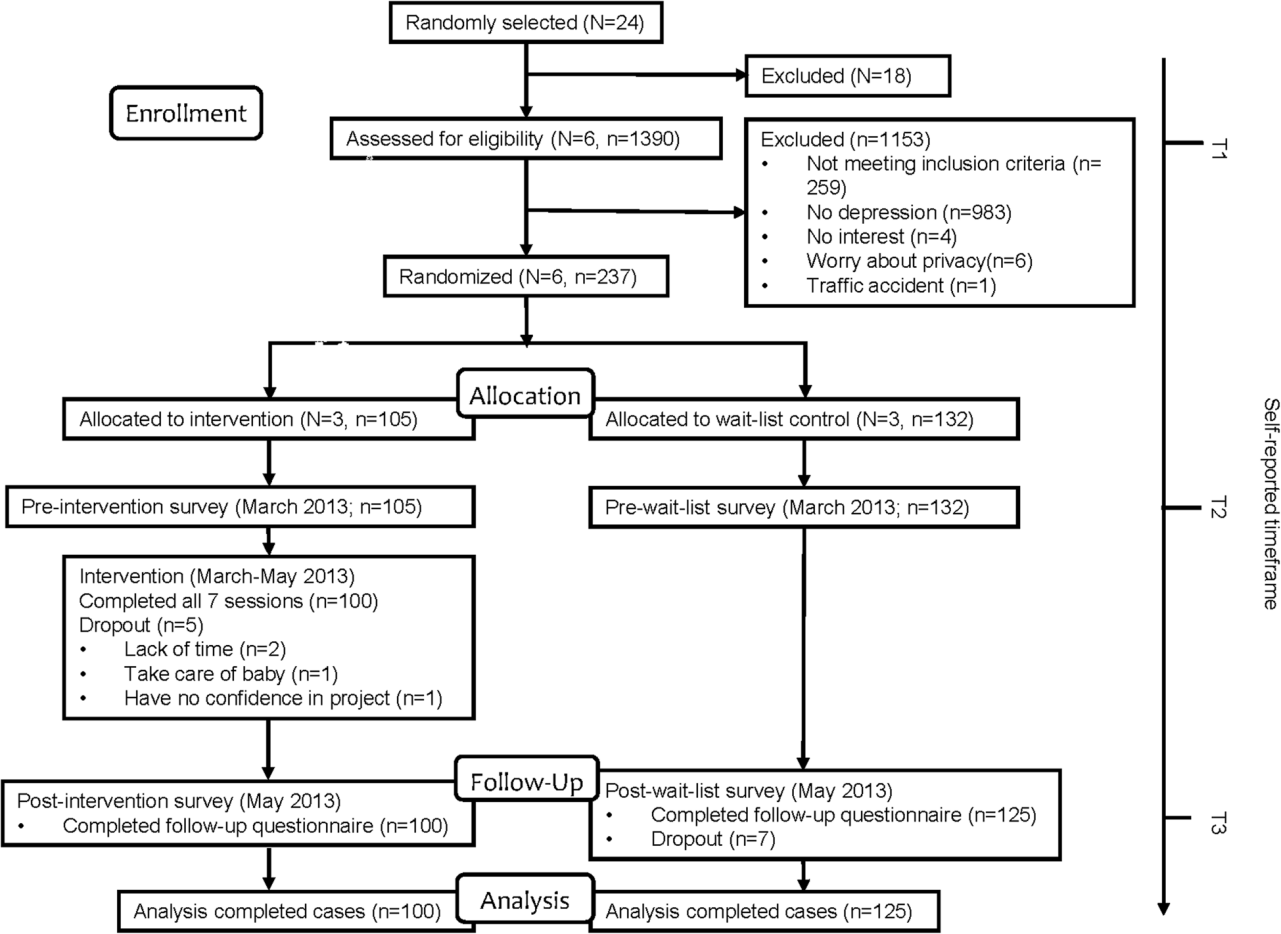
Flowchart of participant’ progress through the trial. The figure shows the study design and procedure. N is the number of communities, and n is the number of residents in the selected communities. Six communities were randomly selected from 24 communities in Pudong District, Shanghai. A depression screening was conducted in 1390 residents at baseline (T1). The intervention group and the wait-list control group were randomized based on the community. The intervention was conducted in the following 2 months after the screening, and 237 individuals participated in the final intervention program, including 105 participants in the intervention group and 132 in the control group. A total of 225 residents completed all the intervention sessions and follow-up survey and entered in the final analysis. The numbers that were lost to follow-up are also shown. Other reasons for loss to follow-up included a lack of time, a lack of confidence in the program, and taking care of a baby

The study used a cluster randomized wait-list controlled design (Fig. 1). Participants were randomized to either the intervention group or to a wait-list control group based on community. A baseline screening survey (T1) was conducted 6 months before the intervention from July to August, 2012. The questionnaires were performed again immediately before the intervention (T2) and immediately after the intervention (T3). Several well-trained research assistants conducted the testing. From March to May of 2013, the intervention group underwent a 2-month mutual recovery program intervention, which included seven 90-min, weekly sessions that were based on a standardized self-designed schedule. A trained instructor with master degree of medicine, who has had experiences with elderly adults, conducted each session. The control group also received the intervention after 1 year on the wait-list (12 months after T3).

### Setting

The program was conducted in communities in Pudong Distric, Shanghai. Pudong District is a deputy provincial municipal district located in the east of Shanghai, having the largest population among regions in Shanghai. Pudong District maintains a steady and relatively fast economic growth level and accounts for almost 1/3 of the total gross domestic product (GDP) of Shanghai.

### Participants

In total, 6 communities randomly selected from 24 communities in Pudong District were invited to participate in the study (Fig. 1).

A depression screening was conducted in 1390 residents from the selected 6 communities at baseline before the intervention. The recruitment team was comprised of our researchers and local health care workers who received specific training. Organization and mobilization were conducted in the following 2 months after screening and a total of 237 residents from 6 communities were recruited to participate in the intervention program, including 105 participants in the intervention group and 132 in the control group.

The inclusion criteria were as follows: (a) age range from 50 to 80, (b) no terminal illness, (c) ability to speak and read Chinese, (d) no severe concurrent psychological or psychiatric disease or other physical disease that impacts physical activities or mental health, (e) no recent travel plans, (f) permanent residents (lived in the community more than 6 months), (g) a diagnosis of depressive symptom based on a GDS-15 score of 5 or more, and (h) absence of cognitive impairment as determined by scores of 8 or higher on the Mental Status Questionnaire (Kahn, 1960).

### Randomization

To avoid contamination between communities and obtain a better study organization, a statistician independent of the study randomized the intervention group and wait-list control group at community level. Each community was assigned a unique number and 3 communities were randomized for the mutual recovery intervention, the other 3 communities for the wait-list control group by Predictive Analytics Software (PASW) 18.0 for Windows Random Sampling.

### Attrition

Five participants (2.11%) from the intervention group dropped out before the intervention was completed and seven participants (2.95%) of the control group did not complete the follow-up questionnaire. Finally, a total of 225 participants who completed all the seven sessions and answered all T1, T2, and T3 questionnaires entered the final analysis. Univariate ANOVAs and chi-square tests showed no differences between the program completers and non-completers (who answered the T1 questionnaire but did not complete all the sessions or not answer the T2 and/or T3 questionnaires) based on demographic characteristics (gender, marriage, age, occupational type, and education background) or the baseline variables. This result suggested that there was no systematic difference in gender, marriage, age, occupation type, and education between those who dropped out and those who didn’t.

### Sample size

The sample size was determined based on the main outcome measures (GDS-15) of previous depression intervention studies, and the effect size of Cohen’s *d* =0.83 [10]. The G*Power 3 program was used to calculate the sample size [27]. We took the intra-cluster correlation coefficient (ICC) into account, assuming a conservative value of intra-cluster correlation of *ρ* =0.05 [28] and 20 participants per cluster (*m*). To allow for a significant level of two-sided *α* =0.05 and 80% power (1-*β*), *n* =171 individuals per arm was required.

As our study had only recruited a limited number of communities and the sample size might not be sufficient according to the design effect size above, we need to reset the minimum detectable difference. With a fixed number of clusters *k* =3 per arm and a fixed average number of individuals per cluster *m* =40, the minimum detectable difference of Cohen’s *d*_*c*_ should be 1.21 [29].

### Procedure

To ensure the effect of the mutual recovery program and to take full advantage of the local resources, the intervention communities were divided into several subgroups, each containing 15 to 20 participants, based on their interpersonal relationships and shared interests. The three intervention communities were divided into 7 subgroups and the three control communities were divided into 6 subgroups. A seminar room was provided for the sessions in each community. To reduce the bias among different subgroups, all the sessions were conducted by the same instructor. The instructor was one of the core member of the research group who developed the self-management handbook of mental health and the intervention schedule. In addition, a local community worker worked with the instructor as the coordinator to arrange the activities and inform and communicate with the participants. We also chose one participant of each subgroup who was relatively outgoing and good at communication as the leader of the subgroup. The responsibilities of the leaders were as follows: (a) organize and encourage the participants to communicate the knowledge and the difficulties in applying the techniques which were obtained from the sessions, (b) inform the participants about the content that would be taught, (c) set an example and encourage interaction whenever the participants were too shy to follow the requirement, (d) conduct a weekly telephone interview and record their questions on the follow-up, and (e) report the participants’ feedback to facilitators and act as a bridge between the facilitators and the participants for a deeper mutual understanding.

A toolkit was provided to the participants and contained the following: (a) a self-management handbook of mental health that was specifically designed for this program and consistent with the intervention session, (b) a notebook to complete their homework and record any questions regarding applying the techniques or knowledge obtained from the sessions, and (c) a follow-up list that was only administered weekly to the leaders of the subgroups. Every participant should sign in before the session to ensure their attendance. If someone was absent, then the leader or the facilitator would phone to remind or prompt him/her.

The intervention sessions were developed based on the self-management handbook of mental health and the previous studies, and they may not have to been standardized but were adaptive as the content was tailored to the specific needs of the different subgroups. Follow-up information was collected weekly through phone interview by the subgroup leaders to record the mental health changes in every participant in the mutual recovery program groups.

### Intervention

The goal of our intervention was to provide effective, interactive knowledge and skills to the participants, help them identify, assess and change their negative thoughts to positive thoughts, increase their social supports, and finally improve their symptoms of depression (Additional files 1 and 2) and it is invention design and there is before [26]. The topics of intervention sessions were as follows: (i) introduction to depression and self-management: a brief self-introduction of the participants, basic knowledge about mental health, especially depression, “destined acquaintance” activities for pairing up with each other, and a session overview; (ii) relaxation techniques: warm-up game and relaxation techniques practice, including progressive muscle relaxation, guided imagery, and meditation; (iii) emotion regulation: acceptance and tolerance of emotions; story sharing; (iv) problem solving: problem solving skills such as seeking social support; recognition, and modification of maladaptive ways of thinking; (v) sleep hygiene: sleep hygiene education, hypnosis training, and experience sharing; (vi) nutrition and exercise: knowledge about physical activity [26], physical relaxation [30], and healthy diet; relationship between nutrition, exercise, and depression; health knowledge competitions and simple exercise trainings, and (vii) booster session: a summary of the sessions, learning experiences sharing, and commendation.

Music: each subgroup had opportunities to select the songs or music of interest, which would be played before each session and during the partner discussion [22, 23, 31]. This can help create a relaxed atmosphere and ease the tension.

Homework assignment: the participants were required to do homework assignment between each session [23]. Homework from the last session as well as any difficulties in the participants’ lives were discussed first before each session, to promote their implementation of the acquired skills into daily life and communication with each other.

Game time: games were arranged in the middle of each session to active the atmosphere, maintain the participants’ excitement about learning, and improve learning effect [32].

Story sharing: a short allegory related to the session topic was arranged for the participants to discuss and interact before the theoretic knowledge was provided. In the process of discussion, the participants might obtain a better understanding of the subsequent theoretic knowledge.

Our sessions adopted special contents and forms based on Chinese culture. For examples, in the session of “nutrition and exercise” we explained the knowledge of nutrition according to the concept of traditional Chinese medicine (TCM) which advocates natural and science; we taught the elderly how to play tai chi, an ancient Chinese exercise, as a simple exercise training; we introduced “zazen meditation” as a form of relaxations; Chinese classical music and folk songs were chosen by the elderly to appreciate before each session and during the discussion. These cultural-specific forms could make the intervention much easier to accept.

### Measures

All data were collected by self-report questionnaires. Scales and questionnaires used in the study were described below for details.

The Geriatric Depression Scale-15 (GDS-15) was used to evaluate the severity of depressive symptom. The GDS-15 contains 15 items that assess depression especially in older adults, using a yes/no answer format. The original GDS had 30 items [33], and the initial validation study indicated high internal consistency with an alpha of 0.94, and high convergent validity as indicated by a correlation of 0.83 on the Hamilton Rating Scale for Depression (HRSD) [34], which suggested a cut-off point of 5, with scores of 5 or higher being indicative of depression [35]. Internal consistency coefficients were measured in this study. Cronbach’s α coefficients were 0.84, 0.72, and 0.77 for T1, T2, and T3, respectively.

The WHO-5 Well-being Index (WHO-5) was used to assess life satisfaction and depression severity. It was initially developed by Bech. P in 1998 [36, 37] and then revised and recommended to assess life satisfaction by the psychology research collaboration center of the World Health Organization. It was translated into Chinese and introduced to China in 2004 with acceptable internal consistency and validity [38]. There are 5 items in this scale, which uses a Likert 6 rating format from 0 to 5, and usually, the respondent would be considered to have a good life satisfaction with scores higher than the cut-off point of 13 [39]. Cronbach’s α coefficients were 0.89, 0.81, and 0.91 for T1, T2, and T3, respectively.

The sleep condition was assessed using the Self-administered Insomnia Questionnaire [40, 41], which uses a Likert 5 rating format from 1 to 5 and includes 3 items that describes 3 aspects of the sleep process: (a) requiring a long time to fall asleep, (b) difficulty staying asleep, and (c) waking up too early. Insomnia was diagnosed when any one of the 3 items was positive. Cronbach’s α coefficients were 0.83, 0.79, and 0.82 for T1, T2, and T3, respectively.

An in-house developed general demographic questionnaire that included information on gender, age, marriage, education, and pre-work position was used to control the general study condition and specify suitable participants should the program to be generalized in the future.

### Data analysis

PASW 18.0 for Windows was used for the data analysis. Baseline characteristics of both intervention group (P-group) and wait-list control group (C-group) were compared and tested with the *t*-test for continuous data and chi-square test for categorical data. In order to test a potential selection bias, we also compared the baseline characteristics between the program completers and non-completers (who answered the T1 questionnaire but did not complete all the sessions or not answer the T2 and/or T3 questionnaires), using T1 data from the intervention group by chi-square tests and univariate ANOVA. In addition, Pearson Chi-square test was performed to compare the two groups on the differences in the classified data from the 3 assessments.

We used mixed model analysis of variance for repeated measures with the restricted maximum likelihood (REML) estimation method to assess the intervention effects. Time and group were included in fixed effects and subject nested within community (cluster) in random effects. When the group × time interaction was significant, time simple effects were evaluated for each group, and then paired *t*-tests for T1 to T2, T1 to T3, and T2 to T3 in each group were performed (pairwise comparison statistical test results were adjusted using the Bonferroni method) [42].

## Results

Table 1 presents the demographic characteristics of the participants in both groups at the time of inclusion to the trial. As shown, there was no significant difference between the intervention group and the control group in terms of gender, marriage, age structure, post-work type, and educational background (*P* > 0.05).

**Table 1:** Baseline characteristics comparison within the groups (*n* = 225)

		P-group (*n* = 100)^a^	C-group (*n* = 125)^a^	χ^2^	*P*
Gender	Male	25 (25.0%)	38 (30.4%)	0.804	0.370
	Female	75 (75.0%)	87 (69.6%)		
Marriage	In marriage	90 (90.0%)	111 (88.8%)	0.804	0.772
	Out marriage	10 (10.0%)	14 (11.2%)		
Age structure	50 ~ 60year	30 (30.0%)	55 (44.0%)	4.796	0.091
	61 ~ 70year	55 (55.0%)	53 (42.4%)		
	71 ~ 80year	15 (15.0%)	17 (13.6%)		
Post-work type	Physical work	81 (81.0%)	96 (76.8%)	0.854	0.445
	Mental work	19 (19.0%)	29 (23.2%)		
Education	9 years or below	87 (87.0%)	105 (84.0%)	0.400	0.527
	Above 9 years	13 (13.0%)	20 (16.0%)		

^a^P-group: program group; C-group: control group

Chi-square testing was conducted to compare difference of classified data between the two groups at the three measurements. Table 2 shows that there was no significant difference between the two groups at T1 and T2 on the measures of sleep, WHO-5, and GDS-15. However, the perceptions of insomnia, low level of wellbeing and depressive symptom in the intervention group at T3 were significantly lower than that in the control group (*P* < 0.001).

**Table 2:** Difference in the classified data from 3 assessments by Chi-square test (*n* = 225)

		Insomnia *n (%)*	*p*	WHO-5 *n (%)*	*p*	GDS-15 *n (%)*	*p*
T1	P-group	82 (82.00%)	0.275	69 (69.0%)	0.974	100 (100.0%)	-
	C-group	95 (76.0%)		86 (68.8%)		125 (100.0%)	
T2	P-group	57 (57.0%)	0.62	76 (76.0%)	0.286	94 (94.0%)	0.43
	C-group	67 (53.6%)		87 (69.6%)		114 (91.2%)	
T3	P-group	28 (28.0%)	<0.001	14 (14.0%)	<0.001	14 (14.0%)	<0.001
	C-group	68 (54.4%)		89 (71.2%)		111 (88.8%)	

Table 3 presents the interventional effects on sleep, well-being and depressive symptom. A repeated-measure, mixed-model ANOVA shows significant group × time interaction on all the three outcomes (*F* = 8.971, *P* < 0.001; *F* = 36.208, *P* < 0.001; *F* = 62.930, *P* < 0.001, respectively). The time simple effects on sleep, well-being and depressive symptom were statistically significant in the intervention group (*F* = 14.452, *P* = 0.003; *F* = 72.642, *P* < 0.001; *F* = 102.947, *P* < 0.001, respectively) and not significant in the wait-list control group. The scores of insomnia and GDS-15 significantly decreased (*t* = 7.056, *P* < 0.001; *t* = 15.371, *P* < 0.001, respectively) and score of WHO-5 significantly increased (*t* = -15.807, *P* < 0.001) from T2 to T3 in the intervention group. However, the scores of the insomnia and GDS-15 in the control group did not significantly change from T2 to T3. This indicated that the mutual recovery intervention had a positive effect. However, the scores of WHO-5 significantly decreased from T2 to T3 in the control group. A possible reason is that the mental health of these high risk populations with depressive symptoms might continue to deteriorate if they lacked access to timely interventions to gain health literacy. And this then decreased people’s subjective well-being. This is a reminder that we should pay more attention to the mental health of this group because their depressive characteristics have already decided that their mental health will continue to deteriorate if we don’t give any help for them.

**Table 3:** Comparison of scores of sleep, WHO-5, and GDS-15 by mixed effects models ANOVA

	T1^a^		T2		T3		Time*group^d^	Time^e^
	Mean^b^	SD	Mean	SD	Mean	SD		
Sleep								
P-group^c^	9.48	(3.10)	9.52	(3.01)	6.99	(2.48)	*F* = 8.971	*F* = 14.452, *P* =0.003
C-group	8.62	(3.34)	8.80	(2.97)	8.23	(2.79)	*P* < 0.001	*F* = 1.199, *P* = 0.302
WHO-5								
P-group	10.63	(3.31)	10.32	(3.55)	18.22	(4.05)	*F* = 36.208	*F* = 72.642, *P* < 0.001
C-group	11.00	(3.24)	11.26	(4.38)	9.91	(5.19)	*P* < 0.001	*F* = 13.589, *P* = 0.015
GDS-15								
P-group	8.10	(2.14)	7.11	(2.21)	2.99	(1.83)	*F* = 62.930	*F* = 102.947, *P* < 0.001
C-group	7.64	(1.77)	7.10	(2.04)	6.39	(2.68)	*P* < 0.001	*F* = 2.993, *P* = 0.139

^a^T1: baseline, T2: before intervention, T3: posttest

^b^Means and standard deviation (SD)

^c^P-group: program group; C-group: control group

^d^ *F* values and *P* values are reported for each primary and secondary outcome based on multilevel mixed model repeated measures ANOVA

^e^Time simple effect on the outcomes based on multilevel mixed model repeated measures ANOVA

**Fig. 2:**
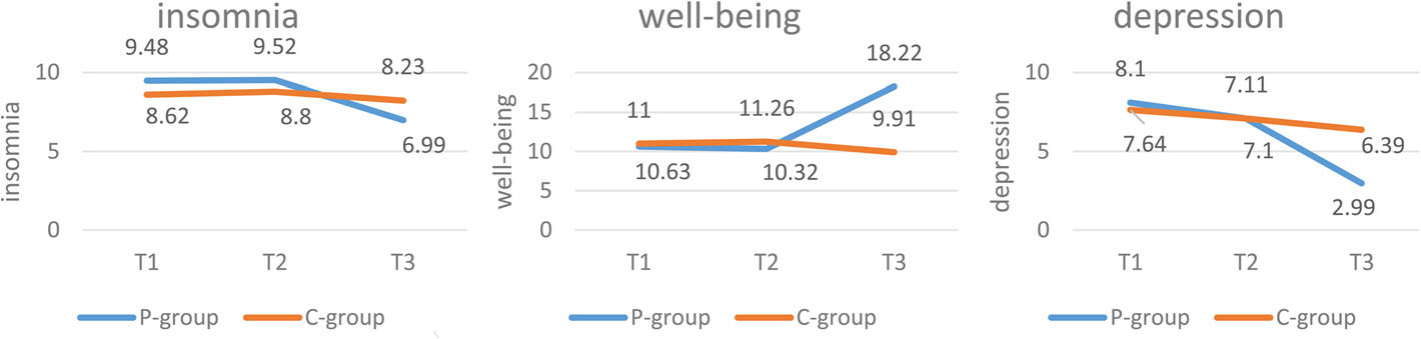
Score changes of Self-administered Insomnia Questionnaire, WHO-5 and GDS-15 on T1, T2, and T3. The figure illustrates the intervention effects on sleep quality, well-being, and depressive symptom. The sample size was 225. See Table 3 for statistical details

Figure 2 intuitively shows the changes in the mean scores of sleep, well-being, and depressive symptom on T1, T2, and T3 in both groups.

## Discussion

The aim of this study was to examine whether a mutual recovery intervention could reduce depressive symptom, and consequently, improve well-being and sleep quality in the older community-dwelling adults with depressive symptom.

The results indicated that the mutual recovery intervention provided effective, interactive skills and improved the participants’ ability to communicate depression-related information and to develop new social connections. Specifically, the program significantly improved the participants’ psychosocial status and living quality such as sleep quality, psychological well-being, and depressive symptom. Psychosocial status and living quality are the core aspects of the expected effect and are required for evaluation by the researchers in the context of the participants’ overall mental health and social situations. In addition, the interaction between the participants and the facilitators can indicate directions for communicating mental health needs and effectively conducting the intervention [16]. This is one of the basic ways that the facilitators who may be unaware of agencies or local mental health resources obtain such information [43]. Therefore, we believe that interaction and communication between participants and facilitators are important parts of the mutual recovery intervention program. Content of every session could be refined before conducting through effective communication with participants. Thus, the program did not simply improve the mental health status of the participants but also taught the facilitators what information or skill the depressive older people need.

The mutual recovery program focused not only on what to teach participants but also on how to organize and present the information and skills. The first step is to gain the trust of the participants before the intervention, no matter how well the program was designed. Music and story sharing constituted our core creative practice to break down social barriers [22, 23] to expressing and understanding experiences and emotions between the participants and the facilitators. These activities can help to create the type of “compassionate” spaces, in terms of mutual, trust, sharing, understanding and recognition that are needed for mental health recovery [13]. Creative practices, such as music, play, and many other forms of art, have so long played a marginal role in many health programs and their supporters and practitioners have often struggled for mainstream jobs [22]. But the facilitators could built trust and deliver the content and techniques more successfully based on this creative practice.

Socioeconomic status is one of the risk factors of depression. People with low income, occupational status, and education tend to be more likely to suffer from depression and other psychiatric disorders than people with higher socioeconomic status [44]. However, this association may not be straightforward. Several researchers have found that the impact of income becomes less crucial beyond the point that people’s income can satisfy their basic material needs [45]. The participants in our study were selected from Pudong District, a relatively rich region in Shanghai and all of the participants lived above the subsistence level. Therefore, we assumed that the economic background of the participants may not exert great influence upon the effect of our intervention in this study. Besides, homogeneity between the two groups was shown because no significant differences occurred between the mutual recovery program group and the control group in terms of gender, marriage, age structure, pre-work type, and educational background. Changes in the two groups of participants provided a rational perspective regarding the benefits of mutual recovery in the older adults. The statistical test results confirmed that the mutual recovery program played a role in increasing the mental health status and meeting the health needs in community-dwelling older adults. Given that the WHO-5 and GDS-15 were used to assess the depression that directly reflects mental health status, the mean scores changed more than that of the SSQ, which was used to evaluate the participants’ psychosocial status. The results indicated that our mutual recovery program was better than the general information dissemination and the participants’ mental health status was improved significantly after the intervention.

The mutual recovery program seems to be a creative and effective way of improving mental health in older community-dwelling adults. However, there still exists an important issue: how can this type of intervention which contains such intensive training and involvement of facilitators disseminate to different communities or different groups of people. The external validity of the results of this study depends on the similarities between the population and intervention of this study and those in the situation of interest. In this study, health care workers from local community health service center attended all the seven sessions and accepted specific training provided by the lecturers of our research team. When the intervention program completed, these health care workers would provide relevant lectures and courses to communities at a frequency of, for example, 4 times a year. Of course, the frequency of the courses may not be so intensive like our study. In addition, the self-management handbook of mental health designed for this program would continue to be distributed to communities for free.

There are some limitations that restrict the wide application of our intervention study. Firstly, to avoid contamination between communities and obtain a better study organization, the study used a cluster randomized controlled design, which ensured the trail scientific and rational and maximized the compliance of the participants and the efficiency and feasibility of the study. That means that we did not randomize the intervention group and the control group by individuals but, rather, by communities. Thus, there may be some confounding factors that we cannot control, such as the economic condition, family construction, and so on. Secondly, cluster trial with a small number of clusters are always controversial, primarily because the small number of units randomized open results to the possibility of bias and approximations to normality become questionable [29]. Limited by funds, labor power, and material resources, we only selected six communities from the 24 communities, and this limited number of clusters and relatively small sample size may bring section bias. However, the results of this study showed that the standardized effect size was about 1.45, which meant that the sample size of this study was sufficient for the design to detect the required difference [28, 29]. In addition, these 6 communities were selected from the same district, and this ensured the homogeneity of their socioeconomic background, which may reduce the bias to some extent. Thirdly, only short-term effects immediately after the intervention were evaluated in this study. Actually, we have followed up the outcomes at 6-, 12-months (immediately before the intervention of the wait-list control group), 14-months (immediately after the intervention of the wait-list control group), and 18-months after the intervention. But this study focused on assessing the effectiveness (whether it is effective or not) of the mutual recovery intervention, rather than the time effect (sustainability of the effect) of the intervention or the changing curve of the effect. So we did not include all of the data. We will analyze the remaining data and report the other consequence in the future. Fourthly, a blind method cannot be used in the intervention because of the process of community mobilization and publicity. Finally, because we cannot intervene in the participants’ daily life, and participants in the control group were not prevented from seeking supplementary help while on the wait-list, contamination of the intervention cannot be fully avoided in this study design.

## Conclusions

The mutual recovery program could effectively improve symptoms of depression, sleep quality and psychological well-being in older community-dwelling adults with depressive symptom. Our findings indicated the importance of communication with facilitators and participants, as well as the interaction between participants and facilitators during the intervention. Further evidence is required to examine the long-term effectiveness of the mutual recovery approach based on the individual level and to promote its further popularization.

## Additional files

Additional file 1: Protocol 1 English translation of trial protocol. (PDF 403 kb)
Additional file 2: Protocol 2 Original Chinese language version of trial protocol. (PDF 258 kb)

## Abbreviations

ANOVA: Analysis of Variance
CBT: Cognitive behavior therapy
GDS-15: The Geriatric Depression Scale 15
HRSD: The Hamilton Rating Scale for Depression
PASW: Predictive Analytics Software
SSQ: The Self-administered Sleep Questionnaire
WHO-5: The WHO-5 Well-being Index
