# Supplementary material for: Effects of a mutual recovery intervention on mental health in depressed elderly community-dwelling adults: a pilot study
Source: BMC Public Health. 2017 Jan 3;17:4. doi: 10.1186/s12889-016-3930-z (PMC5209883; doi:10.1186/s12889-016-3930-z)
Supplement: Additional file 1: — Protocol 1 English translation of trial protocol. (PDF 403 kb) [file 12889_2016_3930_MOESM1_ESM.pdf]

“Physical and mental pleasure”

Effects of a Mutual Recovery Intervention on Mental Health in Depressed Elderly Community-dwelling Adults: a Pilot Study

Abstract

Objective: To examine the effects of mutual recovery program intervention in older adults with high risk of depression at communities in Shanghai, explore suitable approaches to improve mental health of community elders and provide basis for future policy making.

Methods/Design: The study involves a cluster-randomized controlled trial. Recruitment were completed between July 2012 and August 2012. Using a, we randomize 6 communities (N=237) into either the intervention group (3 communities, n=105) or to a wait-list control group (3 communities, n=132). All participants should meet screening criteria of depression, which is defined by The Geriatric Depression Scale (GDS-15). From March 2013 to May 2013, participants in the intervention group will receive a two-month mutual recovery program intervention, including 7 ninety-minutes, weekly sessions, which are based on a standardized self-designed schedule.

Discussion: Mutual recovery is a comprehensive and inexpensive approach for mental health intervention and could be a creative and helpful strategy to promote the mental health in older adults with high risk of depression at communities.

Keywords: Depression; Well-being; Community Intervention; Mental Health; Elderly; Cluster Randomized Trial

Background

The proportion of elder adults (8.3% for 65 and over) are being one of the largest segments of Chinese population. The number has already reached 110 million, which is also the largest in the world [1, 2]. In the meanwhile, as the common disease among older adults which cost almost 80 billion dollars per year in China [3], nearly 10% of community elders and 15% to 25% of hospitalized ones are suffering major depression disorders [4]. And the proportion would reach to 35% if the mild depression was figured in [5]. According to the data of World Health Organization, depression is now the fourth-largest burden on disease cost in the world and will be the second in 2020 [6-9]. Mutual recovery is a comprehensive and inexpensive approach for mental health intervention [10]. Among various kinds of intervention programs which have been used to address the problem, cognitive behavior therapy (CBT) is usually used but results are mixed [11-13]. Conventional CBT is delivered face-to-face, meaning that the limited community resources which need to provide to everyone that would potentially benefit for depression will be less [13, 14]. Although the efficiency of CBT is reliable during face-to-face or group teaching procedures, it lacks of interaction and communication within participants and facilitators [15]. And counseling, an effective treatment for depression in population, is not flexible for older depression as it is inaccessible for the elders and inapprehensible because of the barriers between their generations [16].

Facing the growing burden of depression, innovative paradigms of intervention are needed to overcome the weakness as emerged in previous study [17]. The notion of recovering an initiative life and cultivating positive social and communal connections and relationships and well-being through mutual practices and relationships, and participating is gaining increasing concern and support [18, 19]. Mutual recovery focus on mutuality or reciprocity that would benefit anyone who involves in the process of intervention [19-21]. Participants in mutual recovery group will have chance to interact and communicate the contents taught in the session, as well as any interesting topic out

of the session as deeper and deeper connections would be made during the mutual recovery process and an ongoing self-administration mechanism for depression might be activated [22]. And as many commentators remarked that mental health have often been consolidated in strongly individualistic terms [23], the mutual recovery opens up new possibilities for the goodness by sharing their practices and thoughts. Furthermore, during the communication and interaction with participants, the facilitators also have chances to get the feedback about their intervention and refine the schedule all through the total sessions for a better outcome. In addition, music and story sharing, which create opportunities for breaking silence in a supportive environment, were used as an icebreaking tool for expressing and understanding experiences and emotions and persuading new and better identities and communities [24, 25]. It could be a tool for the facilitator to introduce and start the topic and could be a way to lessen their vigilance [24]. The advantages of the main kind of mental health intervention, like CBT [11], problem solving [26], physical activity [27, 28] and relaxing therapy, were also utilized in the session.

Therefore, we developed a mutual recovery intervention based on culture of China and conducted a series of training courses communities in Pudong, Shanghai. We will examine the outcomes and effects of this kind of intervention among older adults with high risk of depression.

Aims and Hypothesis

The principal research purpose is to examine the effects of mutual recovery program intervention in older adults with high risk of depression at communities in Shanghai. Further, the aim of this study is to explore suitable approaches to improve mental health of community elders and provide basis for future policy making.

Methods

Design overview

The study is a two-armed cluster-randomized wait-list controlled trial. Recruitment will be completed between July 2012 and August 2012. Participants are recruited from six communities, which is randomly selected from 24 communities in Pudong district, Shanghai. In order to organize better and reduce potential confounding factors, each community is assigned an unique number for randomization. Using (Predictive Analytics Software, PASW) 18.0 for Windows Random Sampling, six communities are randomized into to either the mutual recovery intervention or wait-list control group. To ensure the effect of the mutual recovery program and take full advantage of the local resources, intervention communities are divided into several subgroups, each containing 15 to 20 participants, based on interpersonal relationships and shared interests, with two prestigious participants as leaders respectively. The intervention group will receive a two-month mutual recovery program intervention, including 7 ninety-minutes, weekly sessions, which are based on a standardized self-designed schedule. Participants in the wait-list group will also receive the intervention one year later. A baseline survey (T1) will be conducted with both the intervention and wait-list control group 6 months before the intervention. The survey will be conducted again right before the intervention began (T2) and immediately after the sessions (T3). Follow-up information will be collected weekly from the subgroup leaders by phone call to record the mental health change process of every participant in mutual recovery program groups

Population

Participants will be selected by following inclusion criteria: (a) age ranged from 50 to 80; (b) no life-threatening illness; (c) ability to read and self-report; (d) no serious concurrent psychological or psychiatric disease or other physical disease that impact physical activities or mental health; (e) no outing in recent period; (f) a diagnosis of Geriatric Depression Scale 15 scoring 5 or more; (g) absence of apparent cognitive impairment as checked by a score of 8 or higher on the Mental Status Questionnaire

(Kahn, 1960).

Intervention

The intervention group will receive a two-month mutual recovery program intervention, including 7 ninety-minutes, weekly sessions, which are based on a standardized self-designed schedule.

A toolkit will be provide to them, containing:

(a) A note book to do their homework and record any question in utilizing the session skills or contents;

(b) A self-administered mental health manual which is in step with the intervention session;

(c) A follow-up list, which is only given to the leaders of subgroup weekly.

These sessions are assorted with the self-administered mental health manual, and they maybe not on a standardized but flexible fashion depending on the different needs from different subgroups. A seminar room will be provided for the sessions in each community and all subgroup sessions will be conducted by the same facilitator, the core member of designing the self-administered mental health manual and self-designed intervention schedule.

The intervention schedule is detailed below:

(i) Self-introduction; general introduction of mental health, especially depression; activities of "destined acquaintance" to pair up all the group members, session overview.

(ii) Develop a plan of the relaxation exercise; meditate or practice deep breathing; correct the cognitive distortions and fight against negative thinking.

(iii) Emotion Management and problem solving; introduce the technique steps to solve the problem and make concrete plans.

(iv) Self-regulation of Insomnia; introduce the relation between sleep and health; make plans of sleep health.

(v) Diet and health; introduce a balanced diet tailored for the elderly and depression;

(vi) Physical activity and health; make them understand how to exercise scientifically and decide a rationale plan of daily activity.

(vii) Summary and commendation; exchanging and sharing

Music, story, and game are used alternately in the intervention sessions to active atmosphere, promote communication and facilitate practice.

- Music: Each subgroup have chances to select their interested song or music, which will be played before each session and during the partner discussion.
- Homework sharing: to begin each session, homework of the last session should be discussed first is also a process of problem solving.
- Game time: games are arranged in the middle of each session to make participants keep excited in learning.
- Story discussion: short allegory, closely related to the theme that is being taught on each session, is arranged for participants to discuss and interact before theoretic knowledge is conducted. In the process of discussion, participants may get a better understanding of the following topic.

Intervention characteristics

- The toolkit is tailored to the community elderly, meeting the actual needs of them. The content of the handbook is easy understanding and the course is interesting and vivid.
- Participants play a major role in the process of intervention under the teacher's guide.
- Emphasis on homework and experience sharing. To begin each session, homework of the last session should be discussed first is also a process of problem solving.
- Teach through lively activities. For example, "Health ten clap" and "Quick reaction test" and "Ice breaking game" are used to facilitate communication and increase people's interest.

- Phone call following-up. Follow-up information is collected weekly from the subgroup leaders by phone call to record the mental health change process of every participant in mutual recovery program groups
- Evaluation will be conducted during the whole program: six months before intervention, right before intervention and after intervention and weekly following-up through phone call.

Ethical Permission

Written Informed Consent Statement Forms will be obtained from the respondents. The right to withdraw and autonomy of the respondents will be explained. For ethical reasons, after first round of intervention is completed, the mutual recovery intervention will be provided to the wait-list control group. The study has received ethical permission from the ethics committee of School of Public Health of Fudan University, China 51 (IRB00002408&FWA00002399).

Data collection and Measure

A baseline survey (T1) will be conducted with both the intervention and wait-list control group 6 months before the intervention. The survey will be conducted again right before the intervention began (T2) and immediately after the sessions (T3). Follow-up information will be collected weekly from the subgroup leaders by phone call to record the mental health change process of every participant in mutual recovery program groups

- **Demographic characteristics**

Including sex, age, marriage status, previous occupation, educational level and so on.

- **Self-reported health**

Height, weight, blood pressure, general health, self-reported chronic disease, smoking, drinking and physical activity.

- **Geriatric Depression Scale-15(GDS-15)**

GDS-15 is used to evaluate depressive risk severity at baseline. GDS-15 contains 15

items to assess depression especially of older adults, using a yes/no answer format. The original GDS have 30 items and the initial validation study indicated high internal consistency, with alpha of 0.94, and high convergent validity, as indicated by a correlation of 0.83 with Hamilton Rating Scale for Depression (HRSD), which suggested a cut-point of 5, with scores of 5 or higher indicative of depression.

- **WHO5 Well-being Index (WHO5)**

WHO5 is used to assess life satisfaction and depression severity. It is initially developed by Bech. P in 1998, then revised and recommended to assess life satisfaction by the psychology research collaboration center of the World Health Organization. It is translated into Chinese and introduced to China in 2004 with acceptable internal consistency and validity. There are 5 items in this scale, using a likert 6 rating format from 0 to 5, and usually the replier would be located as good life satisfaction when scoring over the cut-point of 13.

- **Self-administered Insomnia Questionnaire (SIQ)**

Sleep condition is assessed by Self-administered Insomnia Questionnaire which uses likert 5 rating format, from 1 to 5, and includes 3 items describing 3 aspects of the sleep process, such as: (a) taking a long time to fall asleep; (b) cannot stay asleep; (c) wake up too early. It would be judged as insomnia when any one of the 3 items is positive.

Self-made general demographic questionnaire, including the information of gender, age, marriage, education and pre-work position, is used to control the general study condition and specify the suitable people if this program is going to be generalized in the future.

Data analysis

PASW 18.0 for Windows will be used for data analysis. Pearson Chi-square test will be conducted to compare the two groups on difference of the classified data on 3 assessments. A multivariate ANOVA will be conducted to explore the differences within groups, at each of the 3 testing points, on the measures of sleep, WHO5 and

GDS-15. A series of repeated measures ANOVAs will be conducted to compare the two groups on all the variables tested across the screening, pretesting and post testing assessments. This approach is flexible and it is possible to model the dependence between observations from the same individual. Multilevel models can be applied to normally distributed and non-normally distributed outcomes; we will choose the most appropriate model based on the data.

Discussion

Mutual recovery is a comprehensive and inexpensive approach for mental health intervention and could be a creative and helpful strategy to promote the mental health in older adults with high risk of depression at communities. Mutual recovery program focused not just only on what to teach participants but also on how to organize and present the information and skills.

The intervention effect may be diluted by participants that are health. Considering the intervention effect, we choose participants that are at high risk of depression. The elderly recruitment for this community-level intervention trial has been successful in identifying a large sample of residents at risk in Shanghai using recruiters drawn from the neighborhoods

During the design of the trial, there are a number of methodological issues that we need to consider. We do not randomize program group and control group by individuals but by communities. Thus, there may be some confounding factors that we cannot control, such as the economy condition, family construction and so on. What’s more, blind method cannot be utilized because of the process of community mobilization and publicity. Since we cannot intervene the participants’ daily life, participants in the wait-list condition are not hindered in seeking supplementary help while on the wait-list, so there may exist contamination confounding in the control group.

Funding

The study was financially supported by the National Science-technology Program for the 11th five-year plan (http://program.most.gov.cn/), project number 2009BAI77B06. The funders had no role in study design, data collection and analysis, decision to publish, or preparation of the manuscript.

Project group and collaborating partners

The project group will consist of

- Chao Wang , School of Public Health, Key Lab of Public Health Safety of the Ministry of Education, Fudan University, Shanghai, 200032, China; National Institute of Occupational Health and Poison Control, Chinese Center for Disease Control and Prevention, Beijing, 100050, China.
- Yu-Jie Hua, School of Public Health, Key Lab of Public Health Safety of the Ministry of Education, Fudan University, Shanghai, 200032, China.
- Hua Fu, School of Public Health, Key Lab of Public Health Safety of the Ministry of Education, Fudan University, Shanghai, 200032, China.
- Long-Feng Cheng, Disease Prevention and Control Center of Wanzhou, Chongqing, 404000, China.
- Wen Qian, School of Public Health, Key Lab of Public Health Safety of the Ministry of Education, Fudan University, Shanghai, 200032, China.
- Jun-Yang Liu, Shanghai Municipal Health Promotion Committee Office, Shanghai, 200002, China; School of Public Health, Key Lab of Public Health Safety of the Ministry of Education, Fudan University, Shanghai, 200032, China.
- Jun-Ming Dai, School of Public Health, Key Lab of Public Health Safety of the Ministry of Education, Fudan University, Shanghai, 200032, China.

Reference

1. Yu yong, TaoLiJian, Yang guo. (2013) China's ageing population and the need of

the public health service. Chinese journal of gerontology 1: 365.
2. Liu Shibao, Zhang rong, Zhang Xiaogong, et al. (2013) China's ageing population and the present situation of health care. Journal of medicine 1: 234.
3. Hu Shaohua. (2005)Social and economic burden of depressive disorders and countermeasures. Chinese journal of preventive medicine 4: 102.
4. Wang Shizhong, Mr Yang, Zhu Xiulin. (2001) Senile depression and related problems. Chinese journal of misdiagnosis in learning 9: 56-58.
5. Charney D S, Reynolds C R, Lewis L, et al. (2003) Depression and Bipolar Support Alliance consensus statement on the unmet needs in diagnosis and treatment of mood disorders in late life. Arch Gen Psychiatry 60: 664-672.
6. Lebowitz B D, Pearson J L, Schneider L S, et al. (1997) Diagnosis and treatment of depression in late life. Consensus statement update. JAMA 278: 1186-1190.
7. Riedel-Heller S G, Weyerer S, Konig H H, et al. (2012) Depression in old age: Challenge for aging societies. Nervenarzt 83:1373-1378.
8. C M. (2002) The global burden of disease and injuries in 1990 (World Health Organization Report). IntSocSci J 1: 287-296.
9. World Health Organization. (2011) Mental health. Available: http://www.who.int/mental_health/management/depression/en/. Accessed 18 June 2011.
10. Paul Crawford, Brian Brown, Victoria Tischler, Charley Baker. (2010) Health humanities: the future of medical humanities? Mental Health Review Journal 3: 4-10.
11. Gould R L, Coulson M C, Howard R J. (2012) Cognitive behavioral therapy for depression in older people: a meta-analysis and meta-regression of randomized controlled trials. J Am GeriatrSoc 60: 1817-1830.
12. Bentall R. (2009) Doctoring the Mind: Why Psychiatric Treatments Fail 2: 181-182.
13. Goldstein L H, Mcalpine M, Deale A, et al. (2003) Cognitive behaviour therapy with adults with intractable epilepsy and psychiatric co-morbidity: preliminary observations on changes in psychological state and seizure frequency. Behav Res

Ther 41: 447-460.

14. Mclaughlin D P, Mcfarland K. (2011) A randomized trial of a group based cognitive behavior therapy program for older adults with epilepsy: the impact on seizure frequency, depression and psychosocial well-being. J BehavMed 34: 201-207.
15. Paul Crawford, L Lewis, B Brown, et al. (2013) Creative practice as mutual recovery in mental health. Mental Health Review Journal 16: 55-64.
16. Miser W F. (2000) Exercise as an effective treatment option for major depression in older adults. J FamPract 49: 109-110.
17. Khoo S. (2012) Re-interpreting the citizen consumer: alternative consumer activism and the rights to health and development. Social Science and Medicine 74: 14-19.
18. Brad O, Leonard AJ, Michelle D, et al. (2009) Increases in Tolerance Within Naturalistic, Intentional Communities: A Randomized, Longitudinal Examination. American Journal of Community Psychology 44: 188-195.
19. Horton-Deutsch S, Mcnelis A, Day P O. (2011) Enhancing Mutual Accountability to Promote Quality, Safety, and Nurses' Recovery From Substance Use Disorders . Archives of Psychiatric Nursing. 25: 445-455.
20. O'Grady C P, Skinner W J. (2012) Journey as destination: a recovery model for families affected by concurrent disorders. Qual Health Res 22: 1047-1062.
21. Riedel-Heller S G, Weyerer S, Konig H H, et al. (2012) Depression in old age: Challenge for aging societies. Nervenarzt 83: 1373-1378.
22. Unutzer J, Katon W, Callahan C M, et al. (2002) Collaborative care management of late-life depression in the primary care setting: a randomized controlled trial. JAMA 288: 2836-2845.
23. Postert C. (2010) Moral agency, identity crisis and mental health: an anthropologist's plight and his Hmong ritual healing. Cult Med Psychiatry 34: 169-185.
24. Spandler H, Stickley T. (2011) No hope without compassion: the importance of compassion in recovery-focused mental health services. J MentHealth 20: 555-

566.

25. Hacking S, Secker J, Kent L, et al. (2006) Mental health and arts participation: the state of the art in England. J R SocPromotHealth 126:121-127.
26. Hopko D R, Funderburk J S, Shorey R C, et al. (2013)Behavioral Activation and Problem-Solving Therapy for Depressed Breast Cancer Patients: Preliminary Support for Decreased Suicidal Ideation. BehavModif 37:747-748.
27. Mura G, Carta M G. (2013) Physical activity in depressed elderly. ClinPractEpidemiolMent Health 9: 125-135.
28. Oh B, Choi S M, Inamori A, et al. (2013) Effects of qigong on depression. Evid Based Complement Alternat Med 1: 8.
