# Supplementary material for: Effects of a mutual recovery intervention on mental health in depressed elderly community-dwelling adults: a pilot study
Source: BMC Public Health. 2017 Jan 3;17:4. doi: 10.1186/s12889-016-3930-z (PMC5209883; doi:10.1186/s12889-016-3930-z)
Supplement: Additional file 2: — Protocol 2 Original Chinese language version of trial protocol. (PDF 258 kb) [file 12889_2016_3930_MOESM2_ESM.pdf]

“身心愉悦”

互助式方法对老年人心理健康干预与评估方案

摘要

目的 了解社区老年人心理健康状况及其影响因素并识别抑郁高危老年人；通过团体互助干预的方法提高社区老年人的心理健康状况，评价干预方案的实施效果及可行性；探索适合我国社区老年人的心理干预模型和方案，为今后的政策制定提供依据。**方法** 选取上海某社区为研究现场，采用整群随机抽样，在知情同意基础上进行调查。运用社会人口学量表、健康自评量表、生活满意度量表、社会支持评定量表、焦虑自评量表、自我管理睡眠问卷和老年人抑郁量表，2012年6-9月在某社区的6个村/居委，对符合纳入标准的研究对象进行问卷调查，建立基线调查数据库。采用整群随机对照滞后干预设计，以横断面调查发现的需要实施重点干预对象，按社区（居委）随机分为干预组与对照组，干预组于2013年3月-5月接受为期两个月的互助式干预培训，并在干预前后分别进行调查，对干预效果进行评估，对照组滞后1年实施干预。互助式干预是一种基于生活社区，以人际相互支持和技能提升为核心，接受干预的参与者有更多的机会去交流和互动，使参与各方均受益的干预方式。课程内容包括如下部分：健康自我管理、放松训练、解决问题方法、情绪管理和调节、睡眠保健、饮食调养、运动调养、预防心情低落。每周干预一次，每次1小时左右。每周课后布置活动作业，前八周课程完成后一周内安排一次电话访谈，后面每两周一次。**讨论** 团体互助式干预是一个富有创造性和有益的干预方式，以提高参与者沟通交流抑郁症相关信息的积极性和开发新的社会关系的能力，增进社区中老年人心理健康。若研究显示干预效果较好，可以为以后的社区中老年人抑郁干预提供数据和资料支持，并向更多的地方推广。

【关键词】 中老年人；抑郁；社区干预；PHQ-9；GDS-15；生活满意度；失眠；社会支持

立题依据（包括国内外研究现状、水平和发展趋势、经济建设和社会发展需求，当前须解决的主要问题、学术价值等，附主要参考文献）。

抑郁症是一种常见的心境障碍，可能的原因众多，以显著而持久的心境低落为主要临床特征，其心境低落与其处境不相称，严重者可出现自杀念头和行为。多数病例有反复发作的倾向，每次发作大多数可以缓解，部分可有残留症状或转为慢性[1, 2]。患抑郁症除了付出严重的感情和社会代价之外，经济代价也是巨大的。据世界卫生组织统计，抑郁症已成为世界第四大疾病，到 2020 年将跃居第二位[3, 4]。迄今为止，抑郁症病因与发病机制还不明确，比较公认的病因假设有：遗传因素、生化因素和心理-社会因素。处于抑郁状态的患者本人承受着精神甚至躯体的极大痛苦，影响患者的生活质量以及家庭和职业能力，并且抑郁症自杀风险很高[5-7]。我们应该重视其危害。

我国以往进行的精神疾病流行病学调查显示，抑郁障碍患病率较低[8]，1982 年和 1993 年两次调查结果均显示，情感性精神障碍患病率低于国外报道水平[3]。北京心理危机研究与干预中心 2009 年调查发现，中国有 17.5%的成年人至少患有一个月的一种或多种精神疾病。根据这项国内进行的最大规模调查的估计，中国大约有 1 亿 7300 万成年人患有某种精神疾病，而其中的 91%大约 1 亿 5800 万人从未接受过专业治疗[9]。我国改革开放的深入及工业化、城市化的发展，都可能对人们的价值观和生活方式造成冲击，以致应激事件增加，导致抑郁障碍患病率呈上升趋势[10, 11]。

2010 年第六次全国人口普查结果表明，60 岁以上老年人口已到达 1.78 亿，占总人口的 13.3%[12]。目前全国 65 岁以上的老年人口数已达一亿之多，占总人口数的 8.3%。由于中国的人口基数较大与其他发展中国家相比老年人口是最多的，在整个世界范围也是最多的[13]。随着步入中老年阶段即将或者面临退休所带来的人际关系、社会环境、生活习惯和社会角色发生巨大转变，同时随着年龄的增大，各种不良生活事件频发，该人群顿感茫然和无所适从，进而产生孤独、寂寞、失落和惆怅等不良心态[14]。这些不良情绪如果不能及时得到疏导，会对中老年人群的心理状态产生严重的负面影响[15-17]。近年来我国人口老龄化程度持续加

深，对于中老年人群心理健康状况的研究也需要同步跟进。抑郁症成为中老年人心理健康的主要危害之一[18]。随着老龄化程度加深，老年人抑郁障碍作为普遍的公共卫生问题日益受到关注[19, 20]。同时，中国每年老年人群疾病与健康支出高达800亿美元，其中10%的社区老年人已及15~25%的住院老年人患有重度抑郁症，如果将轻中度抑郁算进来的话，比例可能达到35%[21]。因此社区中老年人抑郁障碍的预防、筛查以及治疗等技术研究都应紧跟老龄化时代的脚步。

目前有多种抑郁测评量表包括贝克抑郁量表(Beck Depression Inventory-II , BDI-II)，流行病学研究中心抑郁量表（Centre for Epidemiology Studies Depression Scale, CES-D)，老年抑郁量表（Geriatric Depression Scale-15, GDS-15)，医院焦虑与抑郁量表（Hospital Anxiety and Depression Scale, HADS)，一般健康问卷（General Health Questionnaire, GHQ-12）和病人健康问卷抑郁分表（Patient Health Questionnaire-9, PHQ-9)[22, 23]。国外的研究显示筛查老年人心理健康状况 GDS-15 比 GHQ-12 更灵敏，PHQ-9 与 CES-D 相比其优势在于量表比较简短，并且得分计算容易控制[24-26]。同时国内的研究显示 PHQ-9 条目简洁，操作方便，在社区老年人群中应用具有良好的信效度[27]。鉴于老年人抑郁问题的普遍性和隐匿性，急待寻找一种较好的筛查工具对其进行鉴别。

作为社区开展的抑郁干预项目，是采用公共卫生的三级预防策略，立足于一级预防与二级预防，目标人群不只是抑郁症的患者，而是存在明显抑郁症状者，通过干预，可以减少其发展为临床抑郁症的机会。这对于社区中老年抑郁症状的干预就更有现实意义。

在常用的抑郁干预方法当中，认知行为干预被经常使用方法。但常用的认知行为干预，通过医患双方面对面交流的方式进行，意味着能够提供给每个人利于抑郁康复的交流的机会变得更少[28]。这是无法应对有限的心理医生（或咨询师）与大量的需要帮助的中老年抑郁症状阳性者的矛盾的。开发针对人群实施群体干预的适宜技术就显得十分必要。团体培训过程当中如何让认知行为干预更为有效，如何促进参与者之间、参与者与培训者之间的交流和相互促进[29, 30]也是项目面对的挑战之一。同时考虑到对于人群中的抑郁有效的治疗方法对于老年人群是不够灵活的，由于有代沟，对于老年人来说那些方法是不可接受的不相称的[15]。

随着社会抑郁情况的不断严重，我们需要创新性的干预措施来克服传统干预

措施的不足[31]。通过互助式恢复干预，让患者恢复到患病之前的状态，培养他们积极的社会联系和友谊，这样一个趋势已经越来越明显了[32]。互助式干预是一种基于相互关系或相互作用，使参与各方均受益的干预方式[33]。参与到干预中的参与者有更多的机会去交流和互动，在本干预课题中的每一次课程均按照这样的思想去设计，另外一些激发参与者兴趣的话题被引入干预过程中，让所有的参与者能够有更多机会去交流学习抑郁知识[34]。许多学者已经注意到，抑郁的干预大多是固化在了面对面的个体干预中，并没有发掘群体互助式干预的巨大潜力，本课题中的互助式干预是通过系列活动，让参与对象掌握基本的技能，并通过课后的互助小组活动，来强化技能，提供支持。让参与者通过互助共享，学习知识，掌握技能，分享快乐，以增进其身心愉悦感。更重要的是，随着交流和互动的深入开展，干预实施者同样有更多的机会去得到更多的反馈，并以此来完善干预方案，以达到较好的干预效果。干预中运用音乐和故事讨论作为干预前的破冰环节，制造出积极的学习交流互动的氛围[35]。它们也是每次干预的引子，引出每次干预培训的核心内容和重点，帮助被干预者更好的理解每次课程的内容[23]。如认知行为干预、解决问题、身体运动和放松治疗等主流心理健康干预方法的优势也都讲用于本次的互助式干预项目当中[36, 37]。本次研究中的对照组干预是采用常规服务方式，包括基本心理健康教育和寻求医疗服务的方式等。

本次研究选择上海某社区作为社区中老年人抑郁情绪筛查和干预效果评估试点，在该试点应用多种问卷对其中老年人基本健康状况及其相关因素进行调查同时进行抑郁障碍筛查，探讨社区中老年人群抑郁的影响因素，对其预防和治疗提供依据。将抑郁筛查阳性者随机分为对照组和干预组。干预组给予综合的团体互助式干预，探索适合中国社区老年人的心理干预模型及方案评价干预方案的实施效果和可行性。为今后的政策制定提供依据旨在找到一种安全、实用、有效、经济的老年人抑郁干预综合方案。

参考文献

[1] 张明园. 抑郁症是常见病[J]. 中华精神科杂志,2011,44(1):46.

[2] 刘佳莉，苑玉和，陈乃宏. 抑郁症的治疗研究进展[J]. 中国药理学通报,2011,27(9):1193-1196.

[3] 王祖承. 精神病学[Z]. 2. 北京:人民卫生出版社,2002112-125.

[4] 中华医学会精神科分会编. 中国精神障碍分类与诊断标准第 3 版(CCMD-3)[Z]. 济南:山东科学技术出版社,199028-35.
[5] Fischer G, Brunner R, Parzer P, et al. [Depression, deliberate self-harm and suicidal behaviour in adolescents engaging in risky and pathological internet use][J]. Prax Kinderpsychol Kinderpsychiatr,2012,61(1):16-31.
[6] Almeida O P, Pirkis J, Kerse N, et al. A randomized trial to reduce the prevalence of depression and self-harm behavior in older primary care patients[J]. Ann Fam Med,2012,10(4):347-356.
[7] Healey C, Morriss R, Henshaw C, et al. Self-harm in postpartum depression and referrals to a perinatal mental health team: an audit study[J]. Arch Womens Ment Health,2013,16(3):237-245.
[8] 王金荣，王德平，沈渔邨. 中国七个地区情感性精神障碍流行病学调查[J]. 中华精神科杂志,1998,31(2):75.
[9] Phillips M R, Zhang J, Shi Q, et al. Prevalence, treatment, and associated disability of mental disorders in four provinces in China during 2001-05: an epidemiological survey[J]. Lancet,2009,373(9680):2041-2053.
[10] 潘安，王燕芳，于志杰，等. 中国中老年人抑郁症患病率及其南北地域差异研究[Z]. 南京:200640.
[11] 邱婧君. 不同流动状态人群抑郁症患病率及其影响因素[D]. 首都医科大学,2009.
[12] Riedel-Heller S G, Weyerer S, Konig H H, et al. [Depression in old age : Challenge for aging societies][J]. Nervenarzt,2012,83(11):1373-1378.
[13] 于勇杨土保陶立坚. 中国人口老龄化与公共卫生服务的需要[Z]. 2013.33,93-95.
[14] 吴宏. 关于离退休老人与社会、环境及家庭角色转变的思考[J]. 活力,2011(11):25.
[15] 化前珍，郑晓莉，徐莎莎，等. 养老机构老年人抑郁发生率与负性生活事件的相关性[J]. 解放军护理杂志,2011,28(13):14-16.
[16] 严丹君，俞爱月. 老年人焦虑、抑郁和生活满意度及相关性[J]. 中国老年学

杂志,2011,31(10):1847-1848.
[17] 张玲，徐勇，聂宏伟. 2000～2010 年中国老年人抑郁患病率的 meta 分析[J]. 中国老年学杂志,2011,31(17):3349-3352.
[18] 刘艺敏，徐思红，梁丹丹，等. 上海市部分地区中老年人抑郁状况调查[J]. 同济大学学报（医学版）,2006,27(5):82-85.
[19] Matthews S H, Dunkle R E. Lessons from history: Surviving old age during The Great Depression in the United States[J]. J Aging Stud,2013,27(4):464-475.
[20] van den Kommer T N, Comijs H C, Aartsen M J, et al. Depression and cognition: how do they interrelate in old age?[J]. Am J Geriatr Psychiatry,2013,21(4):398-410.
[21] 胡少华. 抑郁障碍的社会经济负担及对策[J]. 中华预防医学杂志,2005,39(4):283-284.
[22] Smarr K L, Keefer A L. Measures of depression and depressive symptoms: Beck Depression Inventory-II (BDI-II), Center for Epidemiologic Studies Depression Scale (CES-D), Geriatric Depression Scale (GDS), Hospital Anxiety and Depression Scale (HADS), and Patient Health Questionnaire-9 (PHQ-9)[J]. Arthritis Care Res (Hoboken),2011,63 Suppl 11:S454-S466.
[23] Vittengl J R, Clark L A, Kraft D, et al. Multiple measures, methods, and moments: a factor-analytic investigation of change in depressive symptoms during acute-phase cognitive therapy for depression[J]. Psychol Med,2005,35(5):693-704.
[24] 徐勇，吴海苏，徐一峰. 病人健康问卷抑郁量表(PHQ-9)在社区老年人群中的应用——信度与效度分析[J]. 上海精神医学,2007(05).
[25] 金涛，陈树林，沈毅，等. 病人健康问卷抑郁量表在社区老年人中应用的信度与效度研究[J]. 浙江预防医学,2011,23(6):27-29, 33.
[26] Milette K, Hudson M, Baron M, et al. Comparison of the PHQ-9 and CES-D depression scales in systemic sclerosis: internal consistency reliability, convergent validity and clinical correlates[J]. Rheumatology (Oxford),2010,49(4):789-796.
[27] Chen S, Chiu H, Xu B, et al. Reliability and validity of the PHQ-9 for screening late-life depression in Chinese primary care[J]. Int J Geriatr Psychiatry,2010,25(11):1127-1133.
[28] Gould R L, Coulson M C, Howard R J. Cognitive behavioral therapy for depression

in older people: a meta-analysis and meta-regression of randomized controlled trials[J]. J Am Geriatr Soc,2012,60(10):1817-1830.
[29] Goldstein L H, Mcalpine M, Deale A, et al. Cognitive behaviour therapy with adults with intractable epilepsy and psychiatric co-morbidity: preliminary observations on changes in psychological state and seizure frequency[J]. Behav Res Ther,2003,41(4):447-460.
[30] Mclaughlin D P, Mcfarland K. A randomized trial of a group based cognitive behavior therapy program for older adults with epilepsy: the impact on seizure frequency, depression and psychosocial well-being[J]. J Behav Med,2011,34(3):201-207.
[31] Khoo S M. Re-interpreting the citizen consumer: alternative consumer activism and the rights to health and development[J]. Soc Sci Med,2012,74(1):14-19.
[32] Olson B, Jason L A, Davidson M, et al. Increases in tolerance within naturalistic, intentional communities: a randomized, longitudinal examination[J]. Am J Community Psychol,2009,44(3-4):188-195.
[33] O'Grady C P, Skinner W J. Journey as destination: a recovery model for families affected by concurrent disorders[J]. Qual Health Res,2012,22(8):1047-1062.
[34] Unutzer J, Katon W, Callahan C M, et al. Collaborative care management of late-life depression in the primary care setting: a randomized controlled trial[J]. JAMA,2002,288(22):2836-2845.
[35] Hacking S, Secker J, Kent L, et al. Mental health and arts participation: the state of the art in England[J]. J R Soc Promot Health,2006,126(3):121-127.
[36] Hopko D R, Funderburk J S, Shorey R C, et al. Behavioral activation and problem-solving therapy for depressed breast cancer patients: preliminary support for decreased suicidal ideation[J]. Behav Modif,2013,37(6):747-767.
[37] Maiera E. Old age depression and its treatment[J]. Psychiatr Danub,2010,22 Suppl 1:S124-S125.
[38] 彭义升，李娟，戴必兵. 北京市高校离退休老人心理健康状况及其相关因素[J]. 中国老年学杂志,2009,29(21):2782-2785.

[39] 刘艺敏，徐思红，梁丹丹，等. 上海市部分地区中老年人抑郁状况调查[J]. 同济大学学报（医学版）,2006,27(5):82-85.
[40] 李迎春，胡传来，陶兴永，等. 中老年人抑郁症状及其相关因素分析[J]. 中国公共卫生,2006,22(4):398-399.
[41] 张玲，姜洪蕾，冯树梅，等. 心理社会因素对离退休中老年人心理健康的影响[J]. 中国老年学杂志,2005,25(12):1447-1449.
[42] 张玲，徐勇，聂宏伟. 2000～2010 年中国老年人抑郁患病率的 meta 分析[J]. 中国老年学杂志,2011,31(17):3349-3352.
[43] Amy Fiske J L W E. Depression in Older Adults[J]. Annu Rev Clin Psychol.,2009(5):363-389.
[44] Gillerot Y, Vanheck C A, Foulon M, et al. Campomelic syndrome: manifestations in a 20 week fetus and case history of a 5 year old child[J]. Am J Med Genet,1989,34(4):589-592.
[45] Smith P D, Sharp S. Health literacy and prescribing: it is time to change old habits[J]. Am Fam Physician,2013,87(11):755-756.
[46] Butterworth P, Gill S C, Rodgers B, et al. Retirement and mental health: analysis of the Australian national survey of mental health and well-being[J]. Soc Sci Med,2006,62(5):1179-1191.
[47] 熊承清，许远理. 生活满意度量表中文版在民众中使用的信度和效度[J]. 中国健康心理学杂志,2009,17(8):948-949.
[48] Zalsman G, Weizman A, Carel C A, et al. Geriatric Depression Scale (GDS-15): a sensitive and convenient instrument for measuring depression in young anorexic patients[J]. J Nerv Ment Dis,2001,189(5):338-339.
[49] Nakata A, Haratani T, Takahashi M, et al. Job stress, social support, and prevalence of insomnia in a population of Japanese daytime workers[J]. Soc Sci Med,2004,59(8):1719-1730.
[50] 肖水源. 《社会支持评定量表》的理论基础与研究应用[J]. 临床精神医学杂志,1994(02).
[51] 云妙英，叶广俊. 吸烟与抑郁[J]. 中国卒中杂志,2008,3(10):756-759.

[52] 范珊红，化前珍，马艳，等. 西安市城市社区老年人抑郁症状发生率及危险因素研究[J]. 护理研究,2007,21(6):483-485.
[53] 杨本付，刘东光，邵光方. 济宁市老年抑郁情绪的现况及其影响因素的探讨[J]. 中国老年学杂志,2000,19(4):195-196.
[54] 邢凤梅，田喜凤，陈长香，等. 离退休老年人抑郁症状及相关因素分析[J]. 护士进修杂志,2004,19(2):114-115.
[55] 段卫东，刘铁榜，胡赤怡，等. 2005 年深圳市抑郁障碍患病率的现况调查[J]. 中华精神科杂志,2010,43(4):211-216.
[56] 胡志，徐晓超，秦侠，等. 合肥市城市社区老年抑郁症患病率调查[J]. 中国初级卫生保健,2004,18(4):30-32.
[57] 陈正英，楚婷，唐莹，等. 湘、鄂、渝、黔四省边区空巢老人健康状况及卫生服务需求的调查[J]. 中华护理杂志,2009,44(8):754-756.
[58] John M C E. Age and Depression[J]. Journal of Health and Social Behavio,1992,33(9):187-205.
[59] Almeida O P, Flicker L, Norman P, et al. Association of cardiovascular risk factors and disease with depression in later life[J]. Am J Geriatr Psychiatry,2007,15(6):506-513.
[60] 彭义升，李娟，戴必兵. 北京市高校离退休老人心理健康状况及其相关因素[J]. 中国老年学杂志,2009,29(21):2782-2785.
[61] 易欢琼，唐济湘，张晋碚，等. 广东高校离退休老人心理状况研究[J]. 中国老年学杂志,1999,19(3):130-131.
[62] 刘红艳. 某干休所老年人失眠状况调查[J]. 中国心理卫生杂志,2006,20(3):170.
[63] 高平，邢凤梅. 日常锻炼对离退休老人抑郁情绪影响的研究[J]. 当代护士（学术版）,2003(11):1-2.
[64] Potts M K. Social Support and Depression[J]. Social Work,1997,42(3):348-360.
[65] 邬沧萍，谢楠. 关于中国人口老龄化的理论思考[J]. 北京社会科学,2011(1):4-8.
[66] 穆光宗，张团. 我国人口老龄化的发展趋势及其战略应对[J]. 华中师范大学

学报（人文社会科学版）,2011,50(5):29-36.
[67] 杨雪，侯力. 我国人口老龄化对经济社会的宏观和微观影响研究[J]. 人口学刊,2011(4):46-53.
[68] 唐荣，苏维. 人口老龄化对医疗卫生的需求及其对策分析[J]. 现代预防医学,2010,37(5):861-863.
[69] 黄成礼，庞丽华. 人口老龄化对医疗资源配置的影响分析[J]. 人口与发展,2011,17(2):33-39.
[70] Lebowitz B D, Pearson J L, Schneider L S, et al. Diagnosis and treatment of depression in late life. Consensus statement update[J]. JAMA,1997,278(14):1186-1190.
[71] Gallacher R. Mental health in older people... This practice profile is based on NS576 Keenan B et al (2011) Promoting mental health in older people admitted to hospitals. Nursing Standard. 25, 20, 46-56.[J]. Nursing Standard,2011,26(13):59.
[72] Cohen G D. The future of being old in America: a mental health perspective[J]. Am J Geriatr Psychiatry,2000,8(3):185-187.
[73] Knight B G, Sayegh P. Mental health and aging in the 21st century[J]. J Aging Soc Policy,2011,23(3):228-243.
[74] Gould R L, Coulson M C, Howard R J. Cognitive behavioral therapy for depression in older people: a meta-analysis and meta-regression of randomized controlled trials[J]. J Am Geriatr Soc,2012,60(10):1817-1830.
[75] Maiera E. Old age depression and its treatment[J]. Psychiatr Danub,2010,22 Suppl 1:S124-S125.
[76] Koenig H G, George L K, Schneider R. Mental health care for older adults in the year 2020: a dangerous and avoided topic[J]. Gerontologist,1994,34(5):674-679.
[77] O'Connor D, Bruner-Canhoto L. Introduction: elder mental health: the next frontier[J]. J Aging Soc Policy,2011,23(3):219-227.
[78] Oh B, Choi S M, Inamori A, et al. Effects of qigong on depression: a systemic review[J]. Evid Based Complement Alternat Med,2013,2013:134737.
[79] Yesavage J A, Brink T L, Rose T L, et al. Development and validation of a geriatric depression screening scale: a preliminary report[J]. J Psychiatr Res,1982,17(1):37-49.
[80] Unutzer J, Katon W, Callahan C M, et al. Collaborative care management of late-

life depression in the primary care setting: a randomized controlled trial[J]. JAMA,2002,288(22):2836-2845.
[81] Friedman B, Heisel M J, Delavan R L. Psychometric properties of the 15-item geriatric depression scale in functionally impaired, cognitively intact, community-dwelling elderly primary care patients[J]. J Am Geriatr Soc,2005,53(9):1570-1576.
[82] van der Waerden J E, Hoefnagels C, Hosman C M, et al. A randomized controlled trial of combined exercise and psycho-education for low-SES women: short- and long-term outcomes in the reduction of stress and depressive symptoms[J]. Soc Sci Med,2013,91:84-93.
[83] Knox M, Lentini J, Cummings T, et al. Game-based biofeedback for paediatric anxiety and depression[J]. Ment Health Fam Med,2011,8(3):195-203.
[84] Mayo-Wilson E, Montgomery P. Media-delivered cognitive behavioural therapy and behavioural therapy (self-help) for anxiety disorders in adults[J]. Cochrane Database Syst Rev,2013,9:D5330.
[85] Miser W F. Exercise as an effective treatment option for major depression in older adults[J]. J Fam Pract,2000,49(2):109-110.
[86] van Marwijk H W, Wallace P, de Bock G H, et al. Evaluation of the feasibility, reliability and diagnostic value of shortened versions of the geriatric depression scale[J]. Br J Gen Pract,1995,45(393):195-199.
[87] Bech P. Quality of life instruments in depression[J]. Eur Psychiatry,1997,12(4):194-198.
[88] Nakata A, Haratani T, Kawakami N, et al. Sleep problems in white-collar male workers in an electric equipment manufacturing company in Japan[J]. Ind Health,2000,38(1):62-68.
[89] Hopko D R, Funderburk J S, Shorey R C, et al. Behavioral activation and problem-solving therapy for depressed breast cancer patients: preliminary support for decreased suicidal ideation[J]. Behav Modif,2013,37(6):747-767.
[90] Spandler H, Stickley T. No hope without compassion: the importance of compassion in recovery-focused mental health services[J]. J Ment Health,2011,20(6):555-566.

研究假设

因果假设

社区中老年人抑郁情绪影响其失眠质量及身心健康。

干预假设

1) 团体互助式干预可以改善社区中老年人抑郁情绪；
2) 团体互助式干预可增进社区中老年人的睡眠质量，改善其身心健康

研究目的

总目的

对社区中老年人的心理健康进行团体互助式干预并评价干预效果，探索适合我国社区老年人的心理干预模型和方案，为今后的政策制定提供依据。

分目标

（1） 了解社区老年人心理健康状况及其影响因素，并识别抑郁高危人群；
（2） 通过团体互助干预的方法提高社区老年人的心理健康状况，评价干预方案的实施效果及可行性；
（3） 探索适合我国社区中老年人的心理干预模型和方案，为今后的政策制定提供依据。

具体干预目标

（1） 通过团体互助式干预提高社区中老年人心理健康；
（2） 通过团体互助式干预提高社区中老年人睡眠质量；
（3） 通过团体互助式干预提高社区中老年人生活满意度。

创新点

1. 教材量身定做。根据前期基线调查报告，复旦公共卫生学院的专业团队参阅 40 余本国内外老年人身心健康促进及干预策略的专业书籍，为小组量身定制《快乐生活自我管理指引》活动手册。同时为其针对性的制定干预方案。

2. 组员在现场干预活动中唱主角。复旦授课老师以认识行为理论为基础从旁进行引导，主要目标是引导组员进行积极有效的参与和互动。让他们身临其境去学习和掌握远离抑郁的技巧，同时配合组员之间的密切交流以及与授课老师的沟通，进而使干预达到更好的效果。

3. 强调家庭作业完成情况的交流。每堂课都布置一定的家庭作业，要求每一位组员在下一次培训时先就家庭作业完成情况进行交流汇报。通过家庭作业的方式让组员在自己的生活当中去实施学到的技巧，在交流过程中活动经验。

4. 导入互动游戏。注重活动的寓教于乐，以“健康十拍手”、“快速反应测试”、“破冰游戏”、“有缘相识”等活动，成为小组活动的有效润滑剂，为促进组员家庭作业的有效完成和生活行为的改变搭建了互督互促的平台。同时也增加了组员继续参与培训的兴趣和积极性。为干预措施的有效进行，提供了有利保障。

5. 特色放松技巧练习。包括肢体放松、呼吸放松、睡眠放松、 冥想放松技巧等。在抑郁干预当中放松是最重要的，采取多形式的放松技巧练习，让组员有效地掌握技巧。并且去切身体会放松的美好。

6. 电话随访。每次干预课程结束之后安排一次电话随访，对组员的具体学习情况进行动态监测，同时可以及时解决他们在生活中实施技巧遇到的问题，也有利于对授课效果的检查，以利于及时对课程不足之处进行调整。

7. 项目评估贯穿始终，在项目开展前进行需求评估，并对项目进行科学的实施过程评估和效果评价

研究方法

研究现场

选取上海某社区为研究现场，采用随机整群抽样，在知情同意基础上进行调查。选择社区的原则是：1）政府有相关政策；2）企业具备进干预项目实施的配合能力；3）社区领导有积极的意向。

研究对象

（1）基线对象：社区中老年人。

研究对象的入选标准：所被选择对象必须是研究现场所在村（居委）的所有在当地居住 6 个月以上的常住居民，年龄限制在 50-80 岁之间，其文化程度应具有小学以上文化程度，或能看书读报。本次调查设计的调查问卷首页设有知情同意书，在知情同意的基础上，本人愿意参与本项目的社区中老年居民，方可被选入。

剔除标准：在该社区为居住满 6 个月以上者，年龄不在此范围内的人群，文盲，有严重疾病无法自主活动者，经过 3 次动员但仍拒绝参与本次调查研究者。

（2）干预对象：有抑郁情绪的社区中老年人

研究时间

2012 年 6 月-2014 年 12 月

研究设计

（1）通过采用随机整群抽样的方式，2012 年 6-9 月对该社区抽取的 6 个村/居委合纳入标准的中老年人群进行问卷调查。

（2）采用整群随机对照滞后干预设计，以横断面调查发现的需要实施重点干预对象，按社区（居委）随机分为干预组与对照组，干预组在 2013 年 3 月-5 月接受为期两个月的互助式干预培训，并在干预前后分别进行调查，对干预效果进行评估，对照组滞后 1 年实施干预。

滞后式干预可以较好的解决参与的研究对象同等受益的医学伦理问题，又可满足科学设计要求有平行对照的问题。所有研究的重点干预对象均可同等受益，而不是仅干预组研究对象获益的问题。

研究内容

调查方法

采用自编一般人口学量表和特定评估量表对纳入研究的中老年人群行问卷调查。

在填写问卷前由经过统一培训的调查员讲解问卷的内容以及注意事项，并且每个调查地点保证 2 名以上的调查员，以及保证同时接受问卷调查的对象不超过 30 人，问卷采用自填方式。

调查工具

1）一般社会人口学特征：包括性别、年龄、婚姻状况（在婚/非在婚）、主要从事职业、文化程度等。

2）健康自评量表：包括身高、体重、血压、健康自评、自报慢性病患病、吸烟、饮酒、运动频率等。

3）生活满意度量表（WHO-5），由五个条目组成，条目按 likert 6 级设定选项，计分 0 分到 5 分，总分 25 分，得分<13 分者为生活满意度较低，得分>=13 分者为生活满意度较高。

4）老年人抑郁简表（Geriatric Depression Scale -15，GDS-15），由 15 个条目组成，总分 15 分，得分<=5 分者为正常，得分>5 分者判断为抑郁；

5)失眠评定采用自我管理睡眠问卷，包含 3 个条目的症描述分别为：①入睡时间过长，②不能保持睡眠状态，③晨醒过早；只要满足一项即判定为失眠。

6)社会支持评定量表（social support revalued scale，SSRS）共 10 个条目，第 1-4，8-10 条：每条只选一项，选择 1，2，3，4 项分别计 1，2，3，4 分；第 5 条分 A，B，C，D 四项计总分，每项从无到全力支持分别计 1-4 分；第 6、7 条如回答"无任何来源"则计 0 分，回答"下列来源"者，有几个来源就计几分。按 likert 4 级设定选项，总分即 10 个条目得分之和；2、6、7 评分之和为客观支持得分；1、3、4、5 评分之和为主观支持得分；8、9、10 评分之和为支持利用度得分。

7）焦虑自评量表（self-rating anxiety scale，SAS），采用 likert4 级评分，主要评定症状出现的频度，其标准为："1"表示没有或很少时间有；"2"表示有时有；"3"表示大部分时间有；"4"表示绝大部分或全部时间都有。20 个条目中有 l5 项是用负性词陈述的，按上述 l～4 顺序评分。其余 5 项（第 5，9，13，17，19）注*号者，是用正性词陈述的，按 4～1 顺序反向计分。SAS 标准分的分界值为 50 分，其中 50—59 分为轻度焦虑，60—69 分为中度焦虑，70 分以上为重度焦虑。

统计学方法

采用 EPIDATA 3.0 软件建立数据库并进行录入；采用 PASW（SPSS18.0）统计软件包进行描述性分析（分类变量采用卡方检验、连续变量采用 t 检验）和多因素非条件 logistic 回归分析，计量资料以（ $x±s$）表示，以 $P< 0.05$ 为差异有统计学意义。

质量控制

本次现场调查研究由上海复旦大学公共卫生学院牵头，张江镇政府以及所在的社区相关工作人员的积极配合，从而从组织上保证该调查的顺利进行。参与项目的所有调查员均为该项目的核心成员，在进行现场调查之前均必须参加统一的培训，了解本次项目的主要内容，学习该研究项目是具体实施方案。同时需掌握现场调查的基本表达和沟通技巧，以及现场调查时的注意事项。每个调查地点要求两个调查员同时在场进行调查，及时对被调查者提出的问题进行解答。在问卷填写完毕之后，调查员现场检查如有漏填或者未完成的问卷可及时让被调查者如实填写。对于阅读能力较差者，要帮助其保质保量的完成问卷。问卷录入时进行双人两次录入，并且进行两份核对。

干预实施的组织

居委卫生干部担任俱乐部负责人，3 个居委的参与者被分成 7 组，每组人数在 15-20 人之间，每组设立组长 1 名。由居委干部负责对调查对象进行动员，邀请其参加培训，并提供书面的项目内容与活动方案的介绍，告知具体的项目安排和时间。复旦大学公共卫生学院的老师对其进行系列的参与式课程培训，同时居委干部到现场配合，干预开始和结束镇健康办领导参与。

系列干预课程内容安排简介

第一课：认识抑郁

为了让组员之间相互认识；了解什么是抑郁，及抑郁可能引起的各种问题，让组员对抑郁有正确的认识；了解其病因和预警信号，让组员知道什么是自我管理，并如何实施。具体安排：

活动 1：组员自我介绍、鉴定出共同的问题（20 分钟）（入场童年）活动 2：有缘相识活动（10 分钟）（音乐:万水千山总是情）

课间休息（5 分钟）

活动 3：认识抑郁（10 分钟）

活动 4：课程概述、任务及其目的意义（5 分钟）

活动 5：什么是自我管理（5 分钟）

活动 6：结束并留家庭作业（5 分钟）

第二课：如何应对抑郁

通过作业汇报交流讨论，分享同伴活动的乐趣；体验放松的乐趣；领悟放松的技巧；制定放松练习计划。具体安排：

活动 1：家庭作业讨论（10 分钟）

活动 2：热身运动-快速反应游戏（10 分钟）

活动 3：想象放松（10 分钟）

课间休息（5 分钟）

活动 4： 克服消极思维模式（15 分钟）

活动 5： 故事讨论（10 分钟）

活动 6： 结束并留家庭作业（5 分钟）

第三课：解决问题技巧

复习情绪管理技巧，了解其掌握情况；认识解决问题的重要性；介绍解决问题的技巧和具体步骤；制定解决问题的行动方案。具体安排：

活动 1：家庭作业讨论（20 分钟）

活动 2：我的五样（10 分钟）

课间休息（5 分钟）

活动 3：解决问题的技巧（30 分钟）

活动 4：结束家庭作业（5 分钟）

第四课：失眠自我调节

复习解决问题技巧，了解其掌握情况；介绍睡眠的相关知识，使之对睡眠有科学的认识；了解和掌握睡眠保健技巧，制定睡眠保健的行动方案。具体安排：

活动 1：家庭作业讨论（10 分钟）

活动 2：睡眠保健（20 分钟）

课间休息（5 分钟）

活动 3：现场催眠（15 分钟）（梦的意识）

活动 4：结束及家庭作业（5 分钟）

第五课：饮食与抑郁

复习睡眠保健技巧，了解其掌握情况；介绍饮食与抑郁的系及其相关知识；抑郁患者了解并掌握如何进行合理膳食；制定适合抑郁的食谱。具体安排：

活动 1：家庭作业讨论（30 分钟）

活动 2：营养知识有奖竞猜（15 分钟）

课间休息（10 分钟）

活动 3：饮食与抑郁（30 分钟）

活动 4：游戏环节（10 分钟）

活动 5：结束及家庭作业（5 分钟）

第六课：运动与抑郁

通过食谱分享，了解其掌握情况；介绍运动与抑郁的关系及其相关知识；抑郁患者了解并掌握如何进行科学运动，制定运动计划。具体安排：

活动 1：家庭作业讨论（30 分钟）

活动 2：运动的意义（15 分钟）

课间休息（10 分钟）

活动 3：如何进行科学的运动（30 分钟）

活动 4：健身十巧手活动（10 分钟）

活动 5：结束及家庭作业（5 分钟）

第七课：交流与分享

对前面所学课程进行总结，交流彼此的学习心得。同时对学习成绩显著者进行表彰。

时间进度

2012 年

- 课题准备及文献查阅，拟定研究方案（已完成）
- 基线调查（正在进行）
- 基线数据整理

2014 年

- 干预前调查（基线后 6 个月）
- 干预组进行第一轮干预
- 干预后第一次调查
- 干预后 6 个月进行第二次随访

2015 年

- 干预后 12 个月进行第三次随访
- 对照组进行第二轮干预
- 干预组进行干预后 14 个月进行第四次随访
- 干预组进行干预后 18 个月进行第五次随访

考核指标和预期产出

考核指标

（1） 了解社区老年人心理健康状况及其影响因素；

（2） 通过团体互助干预的方法提高社区老年人的心理健康状况；

（3） 探索适合我国社区中老年人的心理干预模型和方案，为今后的政策制定提供依据

预期产出

（1） 研究将在核心期刊发表 2-3 篇文章；

（2） 至少作为一名硕士的毕业设计。
